# Electrospun Nanofibers Loaded with Quercetin Promote the Recovery of Focal Entrapment Neuropathy in a Rat Model of Streptozotocin-Induced Diabetes

**DOI:** 10.1155/2017/2017493

**Published:** 2017-01-30

**Authors:** Chonlathip Thipkaew, Jintanaporn Wattanathorn, Supaporn Muchimapura

**Affiliations:** ^1^Department of Physiology and Graduate School (Neuroscience Program), Faculty of Medicine, Khon Kaen University, Khon Kaen 40002, Thailand; ^2^Integrative Complementary and Alternative Medicine Research and Development Center, Khon Kaen University, Khon Kaen 40002, Thailand; ^3^Department of Physiology, Faculty of Medicine, Khon Kaen University, Khon Kaen 40002, Thailand

## Abstract

In this study, quercetin-loaded zein-based nanofibers were developed using electrospinning technique. The therapeutic effect of these quercetin-loaded nanofibers on neuropathy in streptozotocin- (STZ-) induced diabetes in rats was assessed. Diabetic condition was induced in male Wistar rats by STZ, after which a crush injury of the right sciatic nerve was performed to induce mononeuropathy. Functional recovery was assessed using walking track analysis, measurements of foot withdrawal reflex, nerve conduction velocity, and morphological analysis. The oxidative stress status and the ratio of phosphorylated extracellular recognition kinase (pERK)/extracellular recognition kinase (ERK) expression in the nerve lesion were also assessed in order to elucidate the potential mechanisms involved. Results showed that quercetin-loaded zein-based nanofibers slightly enhanced functional recovery from neuropathy in STZ-diabetic rats. The potential mechanism might partially involve improvements in oxidative stress status and the ratio of pERK/ERK expression in the nerve lesion.

## 1. Introduction

Entrapment neuropathy or nerve compression syndrome is a condition caused by a direct pressure on a single nerve. It is very common in people with diabetes. The prevalence of chronic nerve compression in diabetic patients is approximately 33% [1]. It has been reported that nerve susceptibility to compression increases in diabetes. Possible explanations for this phenomenon involve many factors. First, elevation of polyol pathway flux in diabetic condition induces the accumulation of sorbitol, which in turn enhances water content in the diabetic nerve leading to nerve swelling and increased probability for compression at the narrow area [2, 3]. Second, the impairment of protein transport for membrane regeneration at the compression site also occurs as a result of the axonal transport disorder induced by diabetes. In addition, an increase in advanced glycosylation end products in diabetic condition can change nerve properties leading to nerve stiffness [4, 5]. Recent findings also demonstrate that oxidative stress plays a crucial role in nerve damage induced by injury and diabetes [6–8]. Currently, this condition can be treated either by operative or by nonoperative therapies. It has been found that nonoperative treatments, such as steroid injections, provide only a short-term relief for most patients, while long-term relief is achieved rarely in a small percentage of patients [9]. Although operative treatment can be successful, they also carry a risk of surgical errors. Therefore, a novel, effective nonoperative treatment that would also be safe and straightforward is still lacking.

Quercetin (3,3′,4′,5,7-pentahydroxyflavone), a bioflavonoid, provides numerous health benefits. It possesses antioxidant [10], anticancer [11], anti-inflammatory [12, 13], antidiabetic [14], antihypertensive [15], and neuroprotective [16] properties. In addition, oral administration of quercetin to diabetic rats for 6 weeks can ameliorate impaired nerve function in diabetic rats [17]. Recently, transdermal administration of quercetin-loaded nanofibers effectively enhanced memory and improved oxidative stress status in the rat brain [18]. Given the above-mentioned benefits of quercetin and quercetin-loaded nanofibers, their therapeutic potential for nerve damage recovery in diabetic condition, one of the challenges of this decade, has gained attention. Therefore, we aimed to determine the effect of quercetin-loaded nanofibers on functional recovery in streptozotocin- (STZ-) induced diabetes in rats. In addition, potential mechanisms underlying the therapeutic effects of quercetin-loaded nanofibers were also investigated.

## 2. Materials and Methods

Quercetin, streptozotocin (STZ), and zein-purified protein were obtained from Sigma-Aldrich (USA), whereas dimethylformamide (DMF) and sodium acetate were obtained from Merck Chemical Co. (Germany). All other chemicals were in analytical grade and obtained from Sigma-Aldrich (US).

### 2.1. Preparation of Electrospun Nanofibers Loaded with Quercetin

Zein was dissolved in N,N-dimethylformamide (DMF) in a ratio of 1 : 2 (w/v). The solution was slightly stirred for approximately 60 minutes. Then, various concentrations of quercetin were added to produce a spinning solution containing 5, 10, and 15% (w/v) quercetin. The spinning solution was loaded into a plastic syringe connected to a 16-gauge stainless steel needle made at the nozzle. The needle was connected to a high voltage supply (DEL High Voltage 0–100 kV, KKU1 Model). Electric potential value was fixed at 16 kV, and the solution was fed at a rate of 0.2 mL/h using a syringe pump (KKU1 model) during spinning. The electrospinning was carried out at room temperature and collection distance was fixed at approximately 15 cm.

### 2.2. Characterization

#### 2.2.1. Morphology

The morphology of electrospun nanofibers was determined via scanning electron microscopy (SEM, LEO, 1450VP). In brief, a small section of a nanofiber was sputtered with a thin layer of gold prior to SEM observations. The average diameter of the nanofibers was evaluated by randomly measuring nanofiber diameters at 100 different points on SEM images using image analysis software (Proplus Image Tool).

#### 2.2.2. Physical Status and Compatibility

X-ray diffraction analysis (XRD) was carried out by using PANalytical Empyrean X-ray diffractometer (The Netherlands). The XRD patterns were recorded with Cu-*Κα* radiation over the 2*θ* range from 5° to 60° at 45 kV and 40 mA. Attenuated total reflectance Fourier transform infrared (ATR-FTIR) analysis was performed by using FTIR (Tensor 27, Bruker Optics, Germany) over the range of 600–4000 cm^−1^ and at 4 cm^−1^ of resolution.

#### 2.2.3. Determination of Quercetin Release from Electrospun Nanofiber

Zein-based nanofiber mats loaded with 5, 10, and 15% quercetin were immersed in 50 mL of acetate buffer (pH 5.5). The samples were incubated at 37°C and stirred at 100 rpm. Aliquots of each sample (1 mL) were taken from the release medium at specific intervals and diluted to 5 mL with fresh buffer solution to assess the quantity of quercetin released at various times for up to 3 days. Quercetin concentration was measured using colorimetric method.

#### 2.2.4. Animals

Male Wistar rats, 8 weeks old, were derived from the National Laboratory Animal Center, Salaya, Nakhon Pathom. The weight of the animals on the first day of the experiment was 180–220 grams. They were housed 5 per cage and maintained in 12:12 light: dark cycle and given access to food and water ad libitum. All experiments were performed with the approval of the Ethical Committee of the Institution (AEKKU 11/2552) and every effort was made to minimize animal suffering in accordance with the principles for laboratory use and care of European Community (EEC directive of 1986; 86/609/EEC).

#### 2.2.5. Experimental Procedures

Diabetic condition was induced in male Wistar rats by single injection of streptozotocin (STZ) at a dose of 50 mg/kg body weight (BW) via the intraperitoneal (i.p.) route. The rats with plasma sugar levels ≥250 mg·dL^−1^ were regarded as diabetic rats, and they were selected for further studies. Mononeuropathy was induced in diabetic rats by crushing right upper one-third length of the sciatic nerve with hemostatic forceps for 30 s. In brief, the animals were anesthetized by intraperitoneal injection of sodium pentobarbital (50 mg/kg). An incision was made at the back of the thigh, and the right sciatic nerve was carefully exposed at a point immediately distal from the gluteus maximus muscle. The nerve was crushed at a distance of 5 mm distal to the sciatic notch at the same site (mid-thigh) for 30 s using hemostatic forceps, both in the vehicle-treated group and experimental group. The crush injury was performed by the same person in order to minimize the variability in the extent of the nerve injury induced by the crush. Crush lesion was performed to determine the effect of zein-based nanofiber mats loaded with various concentrations of quercetin on the recovery of the sciatic nerve following injury in diabetic rats. Diabetic rats with experimental neuropathy were divided into various groups as follows: (1) DM + crush injury and no treatment, (2) DM + crush injury + zein-based nanofiber mats treatment, (3) DM + crush injury + 5% quercetin, (4) DM + crush injury + 10% quercetin, and (5) DM + crush injury + 15% quercetin. The animals in groups 3–5 were exposed to crush injury and subjected to zein-based nanofibers loaded with quercetin at concentrations of 5%, 10% and 15%. All rats were subjected to the assigned treatments for 21 days. They were assessed for the functional recovery of the sciatic nerve via walking track analysis (De Medinaceli method) and tested for foot withdrawal reflex every 3 days throughout the study period. At the end of the study period, nerve conduction velocity (NCV) was measured. The lesioned nerve was isolated, the axonal density quantified, and oxidative stress status determined including the activities of superoxide dismutase (SOD), catalase (CAT), and glutathione peroxidase (GPx), in addition to the level of malondialdehyde (MDA). On the third and the sixth day of treatment, the lesioned nerves were isolated and the intensity of ERK and pERK1/2 level determined in order to investigate the potential underlying mechanism of action of quercetin-loaded nanofibers.

#### 2.2.6. Assessment of the Sciatic Nerve Conduction Velocity

Conduction velocity of the sciatic nerves was evaluated indirectly by measuring the time required for the supramaximum electrical stimuli to elicit the contraction response of calf muscle innervated by the sciatic nerve. This process was conducted using Power Lab (PowerLab 26T, LTS) at the end of a 21-day treatment period. In brief, the right hind leg of the anesthetized animal (intraperitoneal pentobarbital sodium injection, 50 mg/kg BW) was maintained in full extension using straps. The difference between response times required to elicit muscle contraction after inducing the displacement of stimulation electrode location was recorded. The conduction velocity was calculated by dividing the changes of the distance travelled by the signal between the first and second stimulation (distance changes after changing electrode location) by the changes in the time required to elicit muscle contraction when the electrode location was changed.

#### 2.2.7. Walking Track Analysis

Walking track analysis was performed every 3 days throughout the 21-day experimental period. In brief, paw prints were recorded by soaking the hind paws of each animal with methylene blue and allowing the animals to walk unassisted along an 8.2 × 42 cm walking track that was traced using white paper. Footprints were allowed to dry and the sciatic function index (SFI) was calculated from the footprints according to the following formula:

(1) SFI=−38.3×PLF+109.5×TLF+13.3×ITF−8.8.

[PLF = (EPL − NPL)/NPL, TSF = (ETS − NTS)/NTS, ITF = (EIT − NIT)/NIT. EPL and NPL were experimental and normal print lengths, respectively, whereas, ETS and NTS were experimental toe spreading and normal toe spreading defined as the distances between the first and the fifth digit of both experimental and normal rats. EIT and NIT are experimental and normal intermediate toes spreading, which were the distances between the second and the fourth toe of experimental and normal rats, respectively].

#### 2.2.8. Foot Reflex Withdrawal Test

In this study, the response time of foot withdrawal reflex to temperature stimuli was recorded and used as an index reflecting the recovery of sensory function of the sciatic nerve. Under normal circumstances, healthy rats immediately withdraw their feet after receiving the stimulation, while the animals with sciatic nerve lesion show longer time or fail to produce this response. In brief, the animals were gently placed into a plastic box with a metal floor that was preheated to a certain temperature (hot plate). The time duration between the initiation of the hot plate exposure and the time at which the paw was raised from the floor was recorded and considered as paw withdrawal latency.

#### 2.2.9. Determination of Oxidative Stress Markers

The sciatic nerve was isolated and prepared as homogenate with ten volumes of 0.1 M Tris-HCl buffer (pH 7.4; 10% w/v). Then, nerve homogenate was centrifuged at 2500 rpm at 4°C for 10 minutes. The supernatant was harvested, protein concentration was determined, and oxidative stress markers including malondialdehyde (MDA) levels and the activities of superoxide dismutase (SOD), catalase (CAT), and glutathione peroxidase (GSH-Px) enzymes were measured.

Protein concentration was assessed according to method of Lowry et al. [19]. Bovine serum albumin (BSA) was used as a standard. MDA was measured via thiobarbituric acid reaction (TBAR) [20], whereas determinations of SOD, CAT, and GSH-Px were performed by recording the ability to inhibit cytochrome C, the rate of decrease in H_2_O_2_, and the amount of reduced nicotinamide adenine dinucleotide phosphate (NADPH) oxidized per minute, respectively.

#### 2.2.10. Histomorphological Assessment

At the end of the experiment, the sciatic nerves were rapidly excised at the injury sites and fixed in 4% (vol/vol) formaldehyde for 48 h. The segments were embedded in paraffin; 5 *μ*m sections were prepared from each segment and examined by toluidine blue staining. Five images from each group were randomly selected and analyzed. Density of the myelinated nerve fibers was calculated on the acquired image using Image Proplus.

#### 2.2.11. Determination of Extracellular Signal-Regulated Kinase (ERK) Activation

The lesioned sciatic nerves were isolated and prepared as homogenates using ice-cold homogenization buffer [radio-immunoprecipitation assay (RIPA) buffer with protease inhibitor cocktail]. Nerve homogenate was centrifuged at 14,000*g* for 30 min, and the supernatant was collected. Protein concentration was determined using NanoDrop spectrophotometers. Equal amounts of protein (70 *μ*g) were separated by sodium dodecyl sulfate- (SDS-) PAGE (10% SDS-polyacrylamide gel electrophoresis) and transferred to a polyvinylidene difluoride (PVDF) membrane (Bio-Rad Laboratories, Hercules, CA). After the transfer to the membrane, the prevention of nonspecific binding was achieved by incubating the membrane with 10% dried skimmed milk in Tween buffer (0.05% Tween 20, Sigma) for 1 h at room temperature. The membrane was then incubated overnight either with phospho-ERK1/2 (1 : 1,000, Cell Signaling Technology, Inc., Boston, MA, USA) or with total ERK1/2 (1 : 1,000, Cell Signaling Technology, Inc., Boston, MA, USA). After the incubation, the membrane was subjected to several washing steps. The sections were incubated in HRP-linked secondary antibody (1 : 1,000) for 1 h at room temperature, and signals were visualized by chemiluminescence using enhanced chemiluminescence (ECL) kit (Pierce, Thermo Scientific). The intensity of gray scale images was analyzed using ImageJ software (National Institutes of Health, Bethesda, MD). Data were presented as the intensity of ERK or the ratio of pERK/ERK.

### 2.3. Statistical Analysis

Data were shown as mean ± standard error of mean (SEM). One-way analysis of variance (ANOVA) and post hoc Tukey's test were used as appropriate and *p* value < 0.05 was considered as statistically significant.

## 3. Results

### 3.1. The Morphology of Electrospun Nanofibers

**Figure 1:**
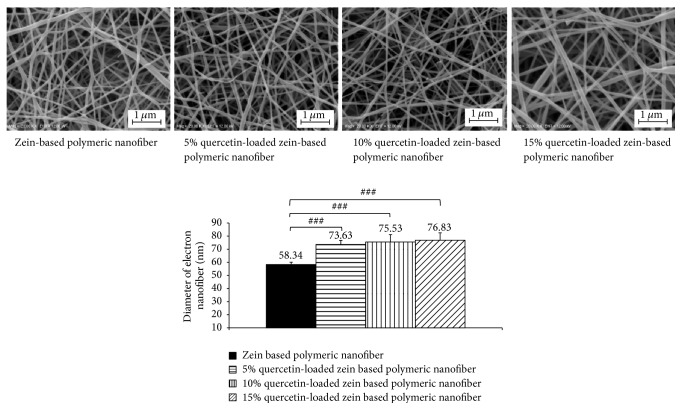
Morphology of zein-based nanofibers and zein-based nanofibers loaded with 5%, 10%, and 15% quercetin visualized by scanning electron microscopy. ^###^*p* < 0.001, compared with zein-based nanofiber.

Figure 1 shows selected SEM images of zein-based nanofiber mats that were nonloaded or loaded with quercetin at concentrations of 5, 10, and 15%. The average diameters of these nanofibers were analyzed using the image analyzing software (Image Proplus, Media Cybernetics Inc.). The diameters of loaded nanofibers were 76.63 ± 3.06 nm, 75.53 ± 5.57 nm, and 76.83 ± 5.67 nm, respectively, whereas the diameter of the nonloaded zein-based nanofiber was 58.34 ± 1.77 nm. Our results clearly showed that the increase of incorporated quercetin concentration in the zein electrospun nanofibers did not produce significant changes in either morphology or diameters.

### 3.2. Physical Status of Quercetin and the Compatibility with Zein Polymeric Nanofiber

**Figure 2:**
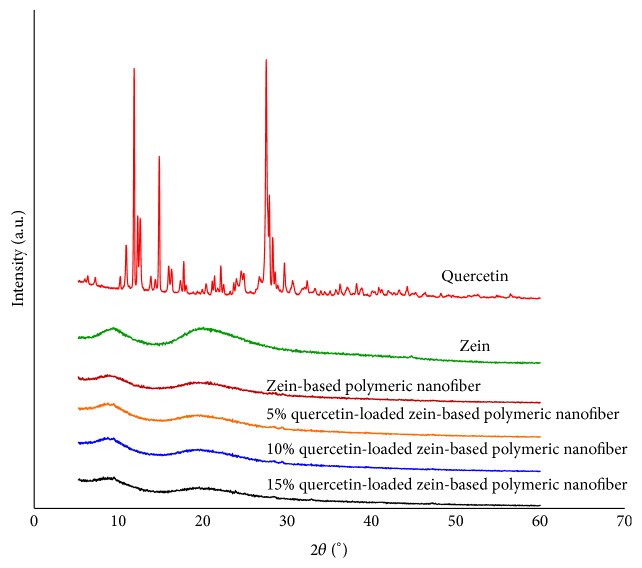
Physical status characterization: X-ray diffraction (XRD) patterns of the raw materials (zein and quercetin) and their polymeric nanofibers; zein-based polymeric nanofibers and quercetin-loaded zein-based polymeric nanofibers prepared by electrospinning.

XRD analyses were carried out to determine the physical status of quercetin in the zein-based polymeric nanofiber.  Figure 2 demonstrates the XRD patterns of quercetin, zein, zein-based polymeric nanofibers, and quercetin-loaded zein-based polymeric nanofibers at the concentrations of 5–15%. XRD pattern of quercetin showed the presence of distinct peaks whereas the XRD pattern of zein nanofiber revealed two broad peaks. The increased quercetin concentrations did not change the XRD pattern of quercetin-loaded zein-based nanofiber.

**Figure 3:**
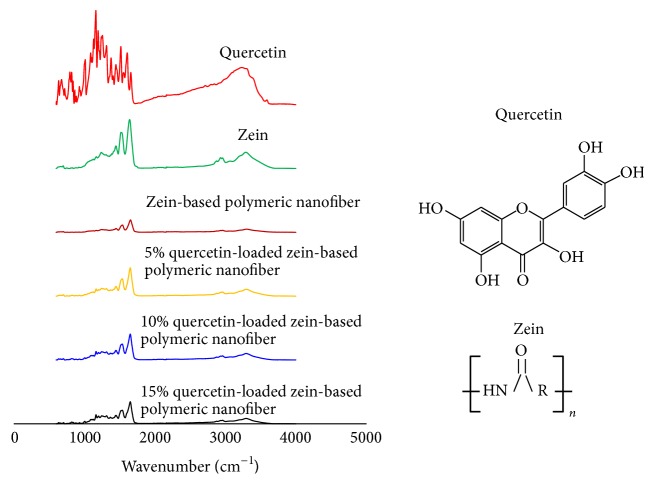
Compatibility investigation: attenuated total reflectance-Fourier transform infrared (ATR-FTIR) spectra of the components (zein and quercetin) and their polymeric nanofibers, zein-based polymeric nanofibers, and quercetin-loaded zein-based polymeric nanofibers.

Figure 3 demonstrates the attenuated total reflectance Fourier transform infrared (ATR-FTIR) spectrum of quercetin, zein, zein-based polymeric nanofiber, 5%, 10%, and 15% quercetin-loaded zein-based polymeric nanofiber. The peaks around 1516, 1607, and 1658 cm^−1^ were observed in the pure crystalline quercetin. It was found that these peaks disappeared when quercetin was loaded to the zein-based polymeric nanofiber. In the finger print region, the peaks of quercetin were shifted and their intensities were attenuated. According to the molecular structures of quercetin and zein in Figure 3, it was found that NH− and −C=O groups in zein could serve as proton donors and proton acceptor for forming hydrogen bonding while free −C=O and −OH groups in quercetin could be served as proton acceptors and donors in hydrogen bonding.

### 3.3. Release of Quercetin from Zein-Based Polymeric Nanofibers Loaded with Quercetin

**Figure 4:**
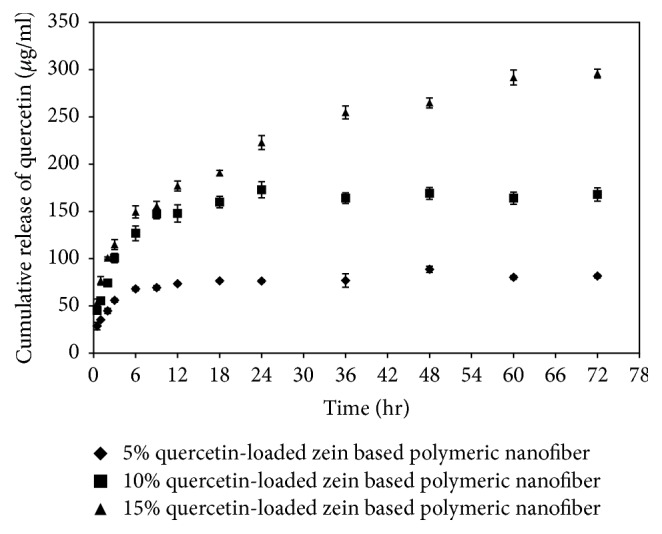
Cumulative release of quercetin from quercetin-loaded zein-based nanofiber mats.

In order to validate that loaded quercetin could be released, we evaluated the dynamics of quercetin release from loaded fibers. Current results show a gradual release of quercetin from 5% quercetin-loaded zein-based polymeric nanofibers that reached a plateau within 24 hours. The releases of quercetin from 10% and 15% quercetin-loaded zein-based polymeric nanofibers were slightly increased and did not show saturation within the 24 hours, as shown in Figure 4.

### 3.4. The Effect of Zein-Based Polymeric Nanofibers Loaded with Quercetin on the Lesioned Nerve Functions in Diabetic Rats

The effects of zein-based nanofibers and quercetin-loaded nanofibers on motor function of the sciatic nerve were assessed using sciatic function index (SFI). This index is based on the crucial role of the sciatic nerve in muscle function, particularly in its weight-bearing support ability that is reflected by changes in footprints. Relevant data are shown in Table 1. It was found that zein-based polymeric nanofiber treatment significantly improved SFI at 9, 12, and 21 days of treatment (*p* value < 0.001, 0.05, and 0.001, resp., compared with diabetic rats with nerve crush injury without treatment). Therefore, our results indicated that even nonloaded nanofiber mat significantly improved SFI. Diabetic rats with nerve crush injury that were treated with zein-based polymeric nanofibers loaded with 5% quercetin showed significant improvement of SFI at 9, 15, and 18 days of treatment (*p* value < 0.01, 0.01, and 0.05, resp., compared with diabetic rats with nerve crush injury that were treated with zein-based nanofibers). Significant improvements of SFI in diabetic rats with nerve crush injury treated with zein-based nanofibers loaded with 10% and 15% were observed at 15, 18, and 21 days of treatment (*p* value < 0.01 all; *p* value < 0.001 all; *p* value < 0.01 and 0.001, resp., compared with diabetic rats with nerve crush injury treated with zein-based nanofibers).

Table 2 shows the effect of zein-based polymeric nanofibers and zein-based polymeric nanofibers loaded with quercetin at various concentrations on latency of foot withdrawal reflex. Our data showed a significantly decreased foot withdrawal reflex latency in diabetic rats with sciatic nerve crush injury that received zein-based polymeric nanofibers at days 6 and 9 of the study period (*p* value < 0.01 all, compared with diabetic rats with nerve crush injury that received no treatment). Diabetic rats with the sciatic nerve lesion that received 5% quercetin-loaded nanofiber mat treatment showed a significant decrease in latencies of foot withdrawal reflex at days 1 and 3 of the study period (*p* value < 0.01 all, compared with diabetic rats with the sciatic nerve lesion that received zein-based nanofibers) whereas those that received 10% and 15% quercetin-loaded polymeric nanofiber mats treatment showed a significant reduction of foot withdrawal reflex latencies only at day 3 during intervention (*p* value < 0.05 and 0.001, resp., compared with diabetic rats with the sciatic nerve lesion that received zein-based nanofibers).

**Figure 5:**
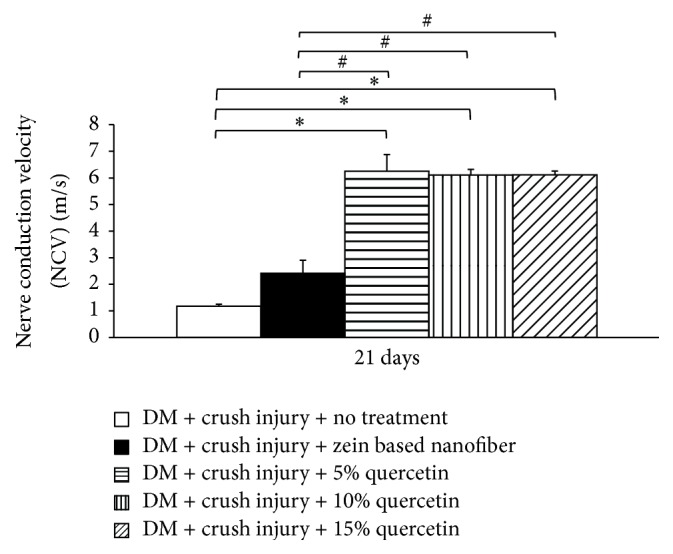
The effect of quercetin-loaded zein-based polymeric nanofiber mats on nerve conduction velocity (NCV) (*n* = 8/group). ^*∗*^*p* < 0.05, compared to diabetes mellitus (DM) + crush injury and no treatment. ^#^*p* < 0.05, compared to DM + crush injury + zein-based nanofiber.

We also investigated the effect of quercetin-loaded zein-based polymeric nanofiber mats on nerve conduction velocity (NCV).  Figure 5 clearly reveals that diabetic rats with the sciatic nerve lesion treated with zein-based polymeric nanofiber mat did not show any significant changes in nerve conduction velocity. Diabetic rats with the sciatic nerve lesion treated with 5%, 10%, and 15% quercetin-loaded zein-based polymeric nanofiber mats treatments showed enhanced conduction velocities (*p* value < 0.05 all, compared with diabetic rats with the sciatic nerve lesion that received zein-based nanofibers).

### 3.5. The Effect of the Zein-Based Polymeric Nanofibers Loaded with Quercetin on Oxidative Stress Status of the Lesioned Nerve in Diabetic Rats

Table 3 shows the effects of quercetin-loaded zein-based nanofibers on oxidative stress markers including MDA levels and the activities of SOD, CAT, and GPx in the lesioned nerve. Diabetic rats exposed to crush injury and receiving zein-based nanofiber mat failed to show significant changes in any of the parameters mentioned earlier. Diabetic rats that were subjected to crush injury and received 5% and 10% quercetin-loaded zein-based nanofibers showed significantly decreased MDA levels (*p* value < 0.01 and 0.05, resp.) compared to the diabetic rats with the sciatic nerve lesion that received zein-based nanofiber mat. However, they also showed significant increases in SOD, CAT, and GPx activities in the lesioned nerve (*p* value < 0.05 and 0.001, resp.; *p* value < 0.05 all; *p* value < 0.05 all) compared to diabetic rats with the sciatic nerve lesion that received zein-based nanofiber mat. However, rats with a 15% quercetin-loaded zein-based nanofibers failed to show significant reduction in MDA level, while an elevation of CAT and GPx activities was observed in diabetic rats with the sciatic nerve lesion (*p* value < 0.05 and 0.01, resp., and *p*-value < 0.05 all, compared with diabetic rats with the sciatic nerve lesion that received zein-based nanofiber).

### 3.6. Histology of the Lesioned Nerve

**Figure 6:**
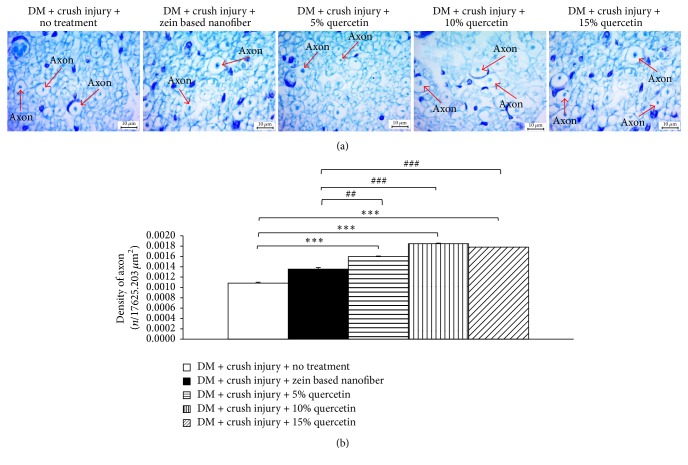
The effect of quercetin-loaded zein-based nanofibers on axonal density. (a) Images showing sections of the lesioned nerve stained with toluidine blue at 100x magnification. (b) Bar graph showing the density of axons in the lesioned nerve in various treatment groups. Values are given as mean ± SEM (*n* = 8/group). ^*∗∗∗*^*p* < 0.001, compared to diabetes mellitus (DM) + crush injury and no treatment. ^##^*p* < 0.05 and ^###^*p* < 0.001, respectively, compared to DM + crush injury + zein-based nanofiber.

The effect of quercetin-loaded zein-based nanofibers on axonal density was also determined, and the results are shown in Figure 6. It was found that zein-based nanofiber mat failed to increase axonal density in diabetic rats exposed to crush injury. Diabetic rats subjected to crush injury and treated with zein-based nanofibers loaded with 5%, 10%, and 15% quercetin showed a significant increase in axonal density (*p* value < 0.01, 0.001, and 0.001, resp., compared with diabetic rats with the sciatic nerve lesion that received zein-based nanofiber).

### 3.7. Effect of Zein-Based Polymeric Nanofibers Loaded with Quercetin on ERK and pERK 1/2

**Figure 7:**
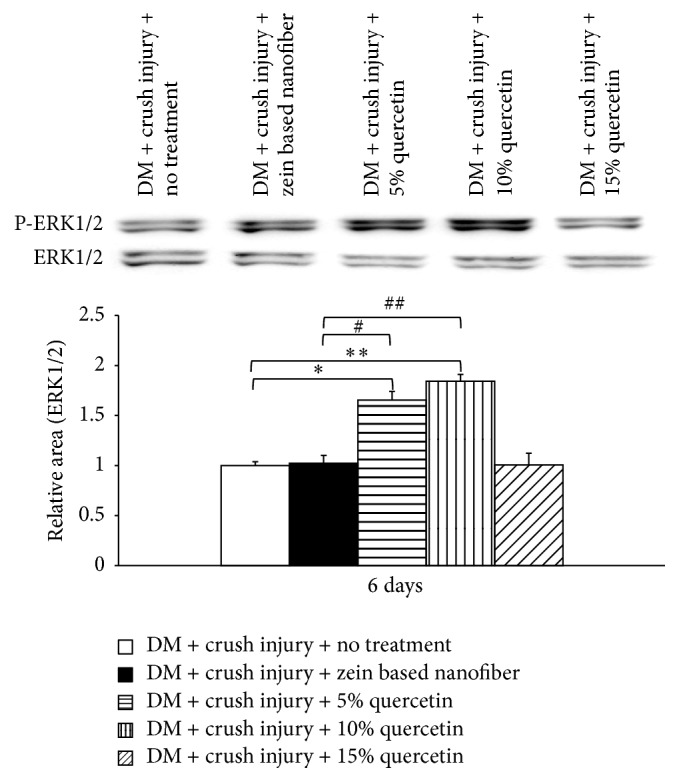
Effect of zein-based polymeric nanofibers loaded with quercetin on levels of extracellular recognition kinase (ERK) and phosphorylated (pERK) 1/2. ^*∗*^*p* < 0.05 and ^*∗∗*^*p* < 0.01, respectively, compared to diabetes mellitus (DM) + crush injury and no treatment. ^#^*p* < 0.05 and ^##^*p* < 0.01, respectively, compared to DM + crush injury + zein-based nanofiber.

Since neurite extension and plasticity of the nervous system have been associated with the function of growth factors and ERK or mitogen-activated protein kinase (MAPK), the effects of quercetin-loaded zein-based nanofibers on modifications of ERK levels in the lesioned nerve were investigated. At day 6 during intervention, diabetic rats with the sciatic nerve lesion that received zein-based nanofibers showed increased levels of phosphorylation of ERK1/2 in the lesioned nerve (Figure 7). Both 5% and 10% quercetin-loaded zein-based nanofibers also produced significant elevation of pERK1/2 levels in the lesioned nerve of diabetic rats (*p* value < 0.05 all, compared with diabetic rats with the sciatic nerve lesion that received zein-based nanofiber).

## 4. Discussion

In this study, we have revealed that quercetin at various concentrations is successfully incorporated into the nanofibers. The efficiency of quercetin loading varied between 63 and 73%. This range is similar to the one used in the studies that loaded nanofibers with other substances, such as Retin-A [21]. XRD pattern of quercetin obtained from this study indicated that quercetin was presented as crystalline material. The disappearance of the diffraction peak of quercetin crystal when quercetin was loaded to the zein-based nanofiber suggested that quercetin in the quercetin-loaded zein-based polymeric nanofibers was changed into an amorphous or noncrystalline form. Based on the molecular structures of zein and quercetin, the interaction between zein and quercetin can also occur [22]. Both the shift and the reduction of all peaks intensities in the finger region of quercetin spectrum also supported that the interaction between quercetin and zein had occurred resulting in the stabilization of environment for the structures and the high degree of compatibility between the components and giving rise to the homogeneous nanocomposite of quercetin and zein [22]. In addition, it has been shown that loaded quercetin can be released from the zein-based nanofiber mat. The release of quercetin from zein-based nanofibers loaded with various concentrations of quercetin is more than 50% within 6 hours and reaches a peak within 24 hours. Then, the capacity for release is maintained until 72 hours. Therefore, these results suggest that quercetin is still released within 72 hours. However, the peak of release is observed within 24 hours. Based on the above-mentioned release characteristics, our in vivo study investigating the effect of quercetin-loaded nanofibers on sciatic nerve functional recovery after injury focused on consequences of daily intervention during a period of 21 days.

Current data clearly shows that quercetin-loaded zein-based nanofibers fully recovered the motor function of the sciatic nerve, which involves large fibers, within the 21-day study period. However, the foot withdrawal reflex in response to heat stimuli, which involves unmyelinated fibers, failed to show full recovery at the end of the study. This indicates that the sensory and motor nerve fibers show different likelihoods of being rescued by quercetin. This was in agreement with a previous study that showed different vulnerabilities between the unmyelinated and myelinated nerve fibers [23, 24]. In addition, the unmyelinated nerve fibers also exhibited slower recovery than myelinated fibers. A possible explanation could be a rapid damage progression of the unmyelinated nerve fiber [23]. Since the unmyelinated nerve fibers experienced more extensive structural damage, they probably required more time for recovery as compared to the myelinated nerve fibers. Therefore, within the duration of our study, diabetic rats with crush injury that received quercetin failed to reach full recovery of sciatic nerve sensory function, whereas their motor functions reflected by a sciatic function index (SFI) showed full recovery. All doses of quercetin loaded on nanofibers also improved NCV and the density of myelinated axons in the lesioned nerve in diabetic rats. Because myelin plays an essential role in determining the speed of nerve conduction, the improvement of NCV observed in this study might be related to enhanced density of myelinated axons in the lesioned nerve.

Several lines of evidence have revealed that oxidative stress also plays a crucial role in nerve destruction after injury [25, 26]. However, our results showed improved oxidative stress status only in diabetic rats with the sciatic nerve lesion that received low and medium doses of quercetin-loaded zein-based nanofibers. These results suggested that other factors influenced functional recovery of the lesioned nerve. Recent findings show that cell survival, signal transduction during myelination, and an establishment of a proregenerative extracellular environment after nerve injury are all under the influence of ERK, a serine/threonine protein kinase [27, 28]. Therefore, the elevation of pERK1/2 levels in diabetic rats with nerve crush injury that received zein-based nanofiber mats loaded with 5% and 10% quercetin observed in this study might play a role in the improvement of nerve regeneration and myelination, which in turn enhanced the recovery of nerve function. In addition, an increase in pERK1/2 levels might have also contributed partially to an increase in axonal density in the lesioned nerve. A lack of correlation between quercetin concentrations and levels of pERK1/2 suggests that other factors might also play a role in the increase of axonal density, and this issue requires further investigation.

## 5. Conclusion

Zein-based nanofiber mats loaded with quercetin enhance functional recovery in experimental diabetic neuropathy. Although modest, this improvement is sufficient to overcome the suffocation and improve the quality of life in these animals. Therefore, this strategy could be used as a tool to facilitate the recovery of nerve function in humans. However, further research is required to explore the effect of quercetin-loaded nanofibers in a more severe injury model and to investigate the underlying mechanism of action in detail.

## Figures and Tables

**Table 1:** The effect of zein-based polymeric nanofibers loaded with quercetin on the lesioned nerve function in diabetic rats (*n* = 8/group). The SFI are regarded as normal when they fall within the range of −10 to 10%. ^*∗*^*p* < 0.05, ^*∗∗*^*p* < 0.01, and ^*∗∗∗*^*p* < 0.001, respectively, compared to DM + crush injury and no treatment. ^#^*p* < 0.05 and ^##^*p* < 0.01, respectively, compared to DM + crush injury + zein-based nanofiber.

Sciatic Function Nerve Index (SFI) at various intervention periods									
Group	Baseline	1 day	3 days	6 days	9 days	12 days	15 days	18 days	21 days
DM + crush injury + no treatment	−8.42 ± 1.23	−93.3 ± 0.00	−93.3 ± 0.00	−93.3 ± 0.00	−93.30 ± 0.00	−70.01 ± 2.70	−62.84 ± 8.78	−57.50 ± 2.74	−37.75 ± 6.65
DM + crush injury + zein based nanofiber	−3.33 ± 6.64	−93.3 ± 0.00	−93.3 ± 0.01	−93.3 ± 0.01	−67.53 ± 5.86^*∗∗∗*^	−63.27 ± 9.46^*∗*^	−53.31 ± 6.32	−44.94 ± 4.62	−16.87 ± 1.58^*∗∗∗*^
DM + crush injury + 5% quercetin	−4.68 ± 7.14	−93.3 ± 0.00	−93.3 ± 0.02	−93.3 ± 0.02	−56.30 ± 2.07^*∗∗∗*,##^	−53.66 ± 9.39^*∗∗*^	−31.49 ± 6.02^*∗∗∗*,##^	−23.53 ± 4.22^*∗*,#^	−9.11 ± 4.66^*∗∗∗*^
DM + crush injury + 10% quercetin	−4.94 ± 3.90	−93.3 ± 0.00	−93.3 ± 0.03	−93.3 ± 0.03	−61.69 ± 2.98^*∗∗∗*^	−56.59 ± 13.17^*∗∗*^	−26.59 ± 3.61^*∗∗∗*,##^	−24.78 ± 3.55^*∗∗∗*,###^	−9.16 ± 1.18^*∗∗∗*,##^
DM + crush injury + 15% quercetin	−8.73 ± 1.13	−93.3 ± 0.00	−93.3 ± 0.04	−93.3 ± 0.04	−93.30 ± 0.00	−78.59 ± 9.11	−30.04 ± 7.64^*∗∗∗*,##^	−26.18 ± 6.45^*∗∗∗*,###^	−8.36 ± 1.26^*∗∗∗*,###^

**Table 2:** The effect of zein-based polymeric nanofibers and zein-based polymeric nanofibers loaded with quercetin at various concentrations on foot withdrawal reflex latency (*n* = 8/group). ^*∗∗*^*p* < 0.01 and ^*∗∗∗*^*p* < 0.001, respectively, compared to DM + crush injury and no treatment. ^#^*p* < 0.05 and ^##^*p* < 0.01, respectively, compared to DM + crush injury + zein-based nanofiber.

Paw withdrawal latency (s.)									
Group	Baseline	1 day	3 days	6 days	9 days	12 days	15 days	18 days	21 days
DM + crush injury + no treatment	2.40 ± 0.58	3.75 ± 0.56	5.09 ± 0.79	4.06 ± 0.83	2.53 ± 0.39	1.75 ± 0.13	1.93 ± 0.09	1.31 ± 0.07	1.25 ± 0.06
DM + crush injury + zein-based nanofibers	2.12 ± 0.18	3.04 ± 0.30	5.18 ± 0.41	2.31 ± 0.25^*∗∗*^	1.78 ± 0.17^*∗∗*^	1.62 ± 0.14	1.62 ± 0.11	1.12 ± 0.06	1.12 ± 0.06
DM + crush injury + 5% quercetin	1.87 ± 0.10	2.76 ± 0.23^*∗∗∗*,##^	2.40 ± 0.24^*∗∗*,##^	1.94 ± 0.19^*∗∗∗*^	2.04 ± 0.29^*∗*^	1.87 ± 0.15	1.87 ± 0.16	1.06 ± 0.04	1.04 ± 0.04
DM + crush injury + 10% quercetin	1.90 ± 0.14	3.15 ± 0.38^*∗∗∗*^	3.34 ± 0.68^*∗*,#^	1.59 ± 0.13^*∗∗∗*^	1.81 ± 0.14^*∗∗*^	1.56 ± 0.10	1.90 ± 0.15	1.03 ± 0.03	1.12 ± 0.06
DM + crush injury + 15% quercetin	1.97 ± 0.00	3.53 ± 0.29^*∗∗*^	2.19 ± 0.21^*∗∗∗*,###^	2.57 ± 0.22^*∗*^	1.96 ± 0.15	1.83 ± 0.25	2.12 ± 0.34	1.53 ± 0.05	1.12 ± 0.06

**Table 3:** The effect of the zein-based polymeric nanofibers loaded with quercetin on the oxidative stress status of the lesioned nerve in diabetic rats (*n* = 8/group). ^*∗*^*p* < 0.05, ^*∗∗*^*p* < 0.01, and ^*∗∗∗*^*p* < 0.001, respectively, compared to DM + crush injury and no treatment. ^#^*p* < 0.05, ^##^*p* < 0.01, and ^###^*p* < 0.001, respectively, compared to DM + crush injury + zein-based nanofiber.

Group	MDA level	SOD activity	CAT activity	GPx activity
	(nmol/min·g. protein)	(u/mg. protein)	(u/mg. protein)	(u/mg. protein)
DM + crush injury + no treatment	0.02 ± 0.005	3.22 ± 0.023	9.87 ± 1.83	9.69 ± 1.17
DM + crush injury + zein-based nanofibers	0.02 ± 0.004	2.75 ± 0.32	7.72 ± 1.24	6.62 ± 1.12
DM + crush injury + 5% quercetin	0.01 ± 0.002^*∗∗*##^	11.77 ± 2.47^#^	21.55 ± 5.23^*∗*#^	24.51 ± 4.12^*∗∗∗*#^
DM + crush injury + 10% quercetin	0.01 ± 0.001^*∗*#^	23.80 ± 2.82^*∗∗∗*###^	23.82 ± 5.56^*∗*#^	24.16 ± 5.09^*∗∗*#^
DM + crush injury + 15% quercetin	0.00 ± 0.000	5.00 ± 0.94	19.74 ± 3.16^*∗*##^	18.22 ± 3.06^*∗*#^
